# CKAP2 Ensures Chromosomal Stability by Maintaining the Integrity of Microtubule Nucleation Sites

**DOI:** 10.1371/journal.pone.0064575

**Published:** 2013-05-30

**Authors:** Chanelle M. Case, Dan L. Sackett, Danny Wangsa, Tatiana Karpova, James G. McNally, Thomas Ried, Jordi Camps

**Affiliations:** 1 Genetics Branch, Center for Cancer Research, National Cancer Institute, National Institutes of Health, Bethesda, Maryland, United States of America; 2 Institute of Biomedical Science, George Washington University, Washington, D. C., United States of America; 3 Section on Cell Biophysics, National Institute of Child Health and Human Development, National Institutes of Health, Bethesda, Maryland, United States of America; 4 Laboratory of Receptor Biology and Gene Expression, Center for Cancer Research, National Cancer Institute, National Institutes of Health, Bethesda, Maryland, United States of America; University of Edinburgh, United Kingdom

## Abstract

Integrity of the microtubule spindle apparatus and intact cell division checkpoints are essential to ensure the fidelity of distributing chromosomes into daughter cells. Cytoskeleton-associated protein 2, CKAP2, is a microtubule-associated protein that localizes to spindle poles and aids in microtubule stabilization, but the exact function and mechanism of action are poorly understood. In the present study, we utilized RNA interference to determine the extent to which the expression of CKAP2 plays a role in chromosome segregation. CKAP2-depleted cells showed a significant increase of multipolar mitoses and other spindle pole defects. Notably, when interrogated for microtubule nucleation capacity, CKAP2-depleted cells showed a very unusual phenotype as early as two minutes after release from mitotic block, consisting of dispersal of newly polymerized microtubule filaments through the entire chromatin region, creating a cage-like structure. Nevertheless, spindle poles were formed after one hour of mitotic release suggesting that centrosome-mediated nucleation remained dominant. Finally, we showed that suppression of CKAP2 resulted in a higher incidence of merotelic attachments, anaphase lagging, and polyploidy. Based on these results, we conclude that CKAP2 is involved in the maintenance of microtubule nucleation sites, focusing microtubule minus ends to the spindle poles in early mitosis, and is implicated in maintaining genome stability.

## Introduction

Chromosome segregation in mitosis is governed by a complex microtubule-based structure arranged in a symmetrical bipolarity with centrosomes located at the two spindle poles. Normally, centrosomes nucleate microtubules that remain anchored to individual spindle poles. With the help of numerous motor proteins and other microtubule-associated proteins, mitotic microtubules become organized between the two centrosomes as a consequence of a minus end-directed microtubule sliding activity present in the spindles [1]. The proper process of distributing the correct number of chromosomes into two daughter cells during mitosis depends on numerous proteins, including centrosomal components and those responsible for the organization of the spindle poles. Often, depletion or malfunctioning of any of these proteins results in the activation of mitotic checkpoints whose function is to arrest cell cycle progression when chromosomes are not properly aligned or attached to the spindles [2].

Typically, microtubules from a single spindle pole attach to individual kinetochores resulting in the equidistant distribution of the metaphase plate. However, recent experiments have shown that most, if not all, lagging chromosomes observed at anaphase are due to merotelic attachments, in which single kinetochores attach to microtubules emerging from different poles [3]. A proposed mechanism of merotelic attachments in cancer is the formation of a multipolar spindle intermediate in cells with multiple centrosomes. In this model, each centrosome forms a spindle pole allowing for greater access of microtubules to kinetochores, which increases the rate of merotelic attachment formation. The supernumerary centrosomes eventually cluster and give rise to a bipolar spindle. However, the aberrant microtubule attachments increase the rate of anaphase lagging chromosomes, and thus are a major cause of aneuploidy. Thus defects in spindle assembly or kinetochore-microtubule attachments can cause aneuploidy, a hallmark of many cancers, in particular of tumors of epithelial origin, i.e., carcinomas [4]. The increased rate of whole-chromosome gains and losses constitutes a phenomenon referred as chromosomal instability [5]. Chromosomal instability frequently correlates with the presence of multiple centrosomes and increased rates of anaphase lagging chromosomes [6], [7]. Ultimately, chromosomal instability seems to enable tumor cells to adapt chromosome content to improve their fitness [8].

Cytoskeleton-associated protein 2 (CKAP2), also known as tumor-associated microtubule-associated protein (TMAP), is frequently upregulated in various malignancies, including stomach cancer and diffuse large B-cell lymphoma [9], [10]. Previous findings suggested that CKAP2 has microtubule-stabilizing properties in interphase cells [11], [12], its degradation is essential for normal completion of cytokinesis [13], and depletion of CKAP2 affects the mitotic process [14]. Phosphorylation of CKAP2 is specific during mitosis and determines the association of CKAP2 with microtubules [15], [16]. It has been suggested that at least four different residues play a role in CKAP2 function in mitosis. Of these, Thr-622 has been shown to be phosphorylated by CDK1-cyclin B1 and to directly regulate spindle dynamics [17]. Recently, Kim et al. [18] showed that CKAP2 is a novel substrate of the Aurora B kinase. Nevertheless, the exact cellular mechanism by which these events occur and the role of CKAP2 in the maintenance of the mitotic spindle and the stability of the genome remain elusive.

In the present study, using the human diploid, karyotypically stable colorectal cancer cell line DLD-1, we elucidated the role of CKAP2 in the formation of the spindle pole, correlated its expression with partners that are known to play a role in the spindle formation, and finally investigated the cellular mechanism by which chromosomal instability arises in cells with altered expression of CKAP2.

## Materials and Methods

### Cell Culture and Synchronization

DLD-1 cells obtained from ATCC (American Type Culture Collection, Manassas, VA) were cultured in RPMI-1640 supplemented with antibiotics and 10% FBS at 37°C in 5% CO_2_. The identity of the cell line was confirmed by the presence of unique chromosomal abnormalities (dup(2p) and t(6;11)) as recorded in the SKY/CGH database (www.ncbi.nlm.nih.gov/sky/) of the National Center for Biotechnology Information/NIH. To enrich for mitotic cells, DLD-1 cells were treated with 100 ng/ml of nocodazole (Sigma-Aldrich, St. Louis, MO) for 16 hours and synchronized in prometaphase. For all immunofluorescence experiments, cells were grown on sterilized 22 mm coverslips inside 6-well plates. Microtubule depolymerization in interphase cells was performed by treating the cells with 10 µg/ml of nocodazole for 30 minutes. Cells were released from the nocodazole block by washing with 1X PBS and incubating in fresh media for the desired time points at 37°C. For the analysis of merotelic attachments, cells were treated with 100 µM monastrol (Sigma-Aldrich) for four hours to accumulate cells in mitosis. Cells were released from the monastrol block by washing with 1X PBS and incubating in fresh media for 45 minutes at 37°C.

### RNAi Experiments

Two different target sequences were selected against CKAP2, siCKAP2_5 (5′-GCA UUU GUU ACU AAC UGA ATT-3′) and siCKAP2_6 (5′-CAC GAU UGU AGA UAU UCU ATT-3′) (Qiagen, Germantown, MD). Additionally, AllStars Negative Control siRNA (scrambled sequence for control), and AllStars Hs Cell Death siRNA (blend of highly potent siRNAs against several mitotic kinases used as a positive control) were used for RNAi experiments (Qiagen). The siRNAs were transfected into 2,500 plated DLD-1 cells at a final concentration of 5nM using Lipofectamine™ RNAiMAX Reagent (Life Technologies, Carlsbad, CA). Target specific transfection efficiency was corroborated at the mRNA level by QRT-PCR and at the protein level by immunoblot.

The target sequence for stably silencing CKAP2 expression using an shRNA expression plasmid was 5′-AGG AAA CAT GTA TTC CTT TAA-3′ (Open Biosystems, Lafayette, CO). Plasmids were transfected into DLD-1 cells using Fugene HD (Promega, Madison, WI). Three days after transfection, positive cells were selected with 2 µg/ml of puromycin (Sigma-Aldrich) for two weeks. In order to enrich for transfected cells, positive cells were separated by fluorescence activated cell sorting (FACS) (FACSCalibur, BD Biosciences, Franklin Lakes, NJ) based on green fluorescence protein (GFP) intensity, regrown in a 100 mm dish, and single-cell sorted into 96-well plates. Each well was individually monitored to ensure that only one cell was plated and single-cell clones were generated accordingly.

### Quantitative Reverse Transcription-PCR

Gene expression levels were assessed using Power SYBR Green technology (Applied Biosystems, Foster City, CA). Gene specific PCR primers were obtained from Operon Technologies, Inc. (Huntsville, AL). The gene YWAHZ was used for normalization. Briefly, five µg of total RNA was reverse transcribed using Superscript II (Life Technologies), the resulting cDNA was diluted 1∶5 and three µl were used in each PCR reaction. PCR was performed with the default variables of the ABI Prism 7000 Sequence Detection System (Applied Biosystems), except for a total reaction volume of 25 µl. Each sample was analyzed in triplicate, and each data point was calculated as the median of the three measured CT values.

### Cytotoxicity Assays: CellTiter-Blue and Annexin V

For the CellTiter-Blue® Cell Viability Assay (Promega), 2,500 cells were transfected with siRNA in 96-well plates and incubated at 37°C for 96 hours. To measure cell viability, transfected cells were incubated with 20 µl of CellTiter-Blue reagent for one hour at 37°C, and fluorescence was measured by SpectraMax M2 (Molecular Devices, Sunnyvale, CA) and analyzed using the software SoftMax Pro (Molecular Devices).

For the Annexin-V staining, 96 hours after siRNA transfection, DLD-1 cells were harvested with CellStripper (Corning, Manassas, VA), washed with binding buffer, and stained with Annexin-V and 7-AAD provided with the Annexin V-PE Apoptosis Detection Kit (BD Biosciences). Transfection with siRNA against PLK1 (Qiagen) was used as a positive control. Stained cells were loaded to FACSCalibur (BD Bioscience) and analyzed by flow cytometry. Apoptotic cells were determined using the software Cell Quest Pro (BD Biosciences) and FlowJo (TreeStar, Inc., Ashland, OR).

### Immunofluorescence

In order to preserve the cellular structure, DLD-1 cells were cultured on a 22 mm coverslip and fixed with a 50∶50 ratio of ice-cold acetone: methanol for 15 minutes at −20°C or 4% PFA with 0.1% Triton X-100 for 15 minutes at room temperature. For analysis of the mitotic spindle, cells were treated with 0.1% Triton X-100 in PHEM for five minutes at room temperature to induce permeabilization. Fixed and permeabilized cells were blocked with 5% BSA with 1% normal goat serum in 0.1% PBST for 30 minutes. Antibodies included: rabbit anti-CKAP2 (Sigma-Aldrich, 1∶100), rat anti-α-tubulin (YL1/2) (Accu-Specs, Westbury, NY, 1∶400) (marker of newly polymerized tubulin, binding to the so called Try-tubulin), mouse anti-α-tubulin, (DM1A) (Sigma-Aldrich, 1∶400) (for total α-tubulin, referred to DM1A in the text), mouse anti-NuMA (BD Biosciences, 1∶100), mouse anti-γ-tubulin (GTU-88) (Sigma-Aldrich, 1∶800), rabbit anti-pericentrin (Abcam, Cambridge, MA, 1∶250), and mouse anti-Hec1 (Abcam, 1∶1,000). Alexa Fluor 488 and Alexa Fluor 568 dyes (Molecular Probes, Life Technologies, 1∶500) were used as secondary antibodies for labeling. Antibodies were diluted in 0.1% PBST with 5% BSA and 1% normal goat serum. The incubation time was overnight at 4°C for primary antibodies and one hour at room temperature for fluorescence-conjugated secondary antibodies. 4,6-diamidino 2-phenyl-indole (DAPI) solution, ProLong Gold Antifade (Life Technologies) was used at the final step for DNA staining. Cells were mounted onto a glass slide for subsequent microscopic observation.

### Nucleation Assay

To depolymerize microtubules, cells were incubated with the microtubule destabilizer nocodazole (10 µg/ml) for 30 minutes. At the end of treatment cells were washed four times with 1X PBS and microtubule re-growth was triggered by transfer to drug-free medium at 37°C. Cells were released for 2, 30, and 60 minutes at 37°C, for a total of one hour release. Slides were then rinsed once in 1X PBS, once with PHEM buffer and then fixed in −20°C methanol. Tubulin structures were detected by incubating cells with a monoclonal α-tubulin (Sigma-Aldrich, 1∶1,000) and rabbit polyclonal γ-tubulin (Sigma-Aldrich, 1∶2,000) antibody at three different time points after drug release.

### Image Processing and Analysis

Images were acquired with the Applied Precision DeltaVision Core System (Applied Precision, Issaquah, WA). This system is based on an Olympus inverted IX70 fluorescence microscope (Olympus America, Inc., Melville, NY) equipped with an automatically controlled stage that allows precise movement in XYZ directions. Data collection is controlled by SoftWoRx software installed on a Linux-based workstation. Images were collected with a 12-bit camera (CoolSnap HQ, Photometrics, Roper Scientific, Tucson, AZ). Cells were examined with a 86000 Sedat Quadruple Filter Set (Chroma Technology Corp, Bellows Falls, VT) which included a FITC filter (Ex 490/20; Em 528/38; Polychroic mirror), a RD-TR-PE filter (Ex 555/28; Em 617/73; Polycroic mirror), and DAPI filter (Ex 360/40; Em 457/50, Polychroic mirror) (Chroma Technology Corp.). Images were analyzed using Metamorph software, version 7.7.4 (Universal Imaging Corporation, Downington, PA). For the microtubule and spindle pole analysis, z-series stacks of images were analyzed and processed using a Delta Vision image processing workstation (Applied Precision). For each slide 200 prometaphase and metaphase cells were analyzed based on microtubule morphology and recorded according to the number of spindle poles with centrosomes and characterized as normal or disorganized. A two-tailed t-test was performed to differentiate between control and CKAP2-depleted cells. Mitotic spindle length was characterized by the distance between spindle poles. A two-tailed t-test was utilized to determine the statistical difference between control and CKAP2-depleted cells. For the total tubulin measurements, 100 cells were analyzed and tubulin measurements were determined by first calculating the total fluorescence in a cell and then subtracting the background intensity from the total intensity. The average intensity and standard deviations were calculated for both control and CKAP2-depleted cells. A two-tailed paired t-test was utilized to determine the statistical difference between the two groups. To analyze merotelic kinetochore orientation in metaphase and anaphase, each slide was analyzed and recorded as normal or as showing lagging chromosomes.

### Immunoblotting

DLD-1 cells were harvested by trypsinization and incubated in SDS lysis buffer (1% SDS, 10 mM Tris-Hcl, pH 7.4, with protease inhibitors). The lysates were sonicated and boiled for five minutes with LDS Sample Buffer (Life Technologies). Protein samples were resolved by 4–12% SDS-PAGE and electroblotted onto a PVDF membrane. The membrane was blocked by soaking in TBS, 0.1% Tween 20, and 5% milk for one hour, incubated with primary antibody with blocking solution overnight at 4°C, washed three times with TBST (TBS, 0.1% Tween 20), incubated with HRP-linked secondary antibodies for one hour at room temperature, and washed three times with TBST. The antibodies used were mouse anti-CKAP2 (Abcam, 1∶1,000), rabbit anti-CKAP2 (Sigma-Aldrich, 1∶1,000), rat anti-α-tubulin (YL1/2) (Accu-Specs, 1∶1,000), mouse anti-phospho-histone H3 (Ser10, 6G3) (Cell Signaling, Danvers, MA, 1∶1,000), mouse anti-cyclin B1 (V152) (Cell Signaling, 1∶2,000), α/γ-tubulin (Cell Signaling, 1∶2,000), GAPDH (Sigma-Aldrich, 1∶40,000), and NuMA (BD Biosciences, 1∶2,000). Anti-mouse IgG, HRP linked, anti-rabbit IgG, HRP linked, and anti-rat IgG, HRP linked antibodies (Cell Signaling) were used for secondary labeling. For the detection of signals, Super Signal West Pico (ThermoScientific, Rockford, IL) was used according to the manufacturer’s recommendations. Films were developed on a Kodak X-OMAT 2000A device.

### Spindle Binding Assay

Cells were arrested in mitosis as previously described (see section cell culture and synchronization). Mitotic cells were harvested by mitotic shake-off and re-suspended in a hypotonic lysis buffer (1 mM MgCl_2_, 2 mM EGTA, 20 mM Tris-HCl, pH 6.8, 0.5% NP-40, 3 µM of taxol, 10 µM of trichostatin A, and protease inhibitors). The lysate was then centrifuged at 15,000 g for one minute to separate the mitotic spindle from the remainder of the lysate. The supernatant and pellet (10 µl per each fraction) were analyzed by Western blot.

### Metaphase Harvesting and Spectral Karyotype (SKY)

Metaphase chromosomes for SKY were prepared after exposure of the control and CKAP2 depleted DLD-1 cells to colcemid (Roche, Indianapolis, IN) for 1–1.5 hours at a final concentration of 0.1 µg/ml. The cells were lysed in hypotonic solution (0.075 M KCl), and the nuclei were fixed in methanol and acetic acid (3∶1). SKY was performed as previously described [19]. For protocol details, please refer to Padilla-Nash et al. [20]. Differentially labeled chromosome-specific painting probes were hybridized simultaneously onto metaphase chromosomes. Images were acquired with a custom-designed triple-pass filter using the SpectraCube SD200 (Applied Spectral Imaging, Carlsbad, CA) connected to an epifluorescence microscope (DMRXA, Leica Microsystems, Wetzlar, Germany). For each clone, at least 20 metaphases and corresponding inverted DAPI images were analyzed with the SkyView software package (Applied Spectral Imaging) and karyotypes were defined using cytogenetic standard nomenclature rules.

At least 100 metaphases previously stained with DAPI were assessed to investigate the variability in the number of chromosomes.

### Live-cell Imaging

DLD-1 cells transfected with empty vector control and CKAP2-depleted cells, were then co-transfected with H2B-Cherry (gifted from Dr. J. Silvio Gutkind, National Institute of Dental and Craniofacial Research, NIH) and selected with geneticin (G418) (Sigma-Aldrich) at a final concentration of 200 µg/ml. Positively selected cells were grown on glass chamber slides (2-chamber) for 72 hours and then analyzed on the Zeiss LSM 5 Live confocal microscope (Carl Zeiss, Inc., Oberkochen, Germany) within an incubation chamber XL LSM710 S1 (PeCon GmbH, Germany) with a heating insert P-LabTek S1. Lasers used: Diode 25 mW 405 nm, DPSS 40 mW 561 nm, Diode 100 mW 488 nm. Time-lapse images were taken from three regions for each sample every three minutes for 72 hours. Maximum intensity projections were taken for each sample and analyzed by ImageJ 1.46.

## Results

### CKAP2 Accumulates in Mitosis and Localizes to the Spindle Pole

**Figure 1:**
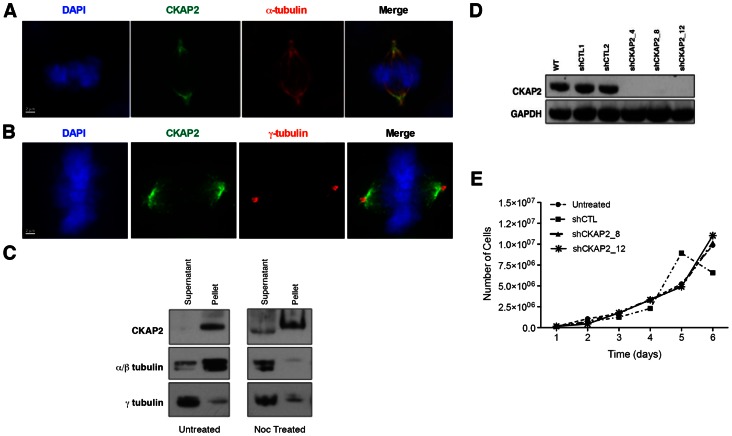
Localization of CKAP2 at the spindle pole in human colorectal cancer cell line DLD-1. (A) DLD-1 cells co-immunostained with CKAP2 (green), α-tubulin (red), and DAPI (blue) depicting localization of CKAP2 to the spindle pole (Scale bar: 2 µm) (B) DLD-1 cells co-immunostained with CKAP2 (red), γ-tubulin (green) and DAPI (blue) demonstrating CKAP2 does not localize within the centrosome (Scale bar: 2 µm). (C) Mitotic cells were enriched by mitotic shake-off, lysed in hypotonic buffer, and the lysate fractionated by centrifugation. The pellet contains DNA, microtubules, and microtubule-associated proteins, whereas the supernatant contains the remaining proteins. These fractions were analyzed by immunoblot with antibodies specific to CKAP2, α/γ-tubulin, and γ-tubulin. The presence of CKAP2 in the pellet in both wild-type and nocodazole treated cells suggests that CKAP2 is indirectly associated with the mitotic spindle. (D) DLD-1 cells were transfected with shRNA, selected with puromycin, and single-cell separated by FACS based on GFP-positivity. Separated cells were synchronized overnight with nocodazole and harvested for immunoblot analysis with antibodies specific for CKAP2 and GAPDH. (E) To measure the affect of CKAP2 reduction on cell proliferation, populations of control (shCTL) and CKAP2-depleted cells (shCKAP2) were counted for six days and plotted. No significant differences in growth activity are observed.

Previous studies in HeLa and HEK293 cells have demonstrated that ectopic expression of CKAP2 is cell cycle dependent, with maximum expression in the G2/M-phase [13], [21]. This expression pattern was confirmed for endogenous CKAP2 in DLD-1 cells (Figure S1). CKAP2 has been shown to associate with microtubules, thus we sought to confirm the subcellular localization of CKAP2 by immunofluorescence. We were able to show that CKAP2 does localize to the mitotic spindle (Figure 1A), and that, despite confinement to the spindle poles, it does not overlap with the centrosome (Figure 1B), as shown by lack of co-localization with γ-tubulin. Furthermore, to determine whether CKAP2 is indeed associated with microtubules, a microtubule-binding assay was performed. By Western blot analysis, we showed that CKAP2 is present in the spindle fraction of the lysate, in both untreated and nocodazole-treated cells (Figure 1C). Altogether, while immunostaining clearly shows the association of CKAP2 with the spindle pole (Figure 1A and 1B), the microtubule-binding assay demonstrates that CKAP2 may not bind directly to microtubules and behave as a canonical microtubule binding protein (MAP). Hence, the association is either mediated by a spindle element distinct from microtubules, or CKAP2 is in a large complex whose association is not microtubule-dependent. Nevertheless, our data suggest that CKAP2 may be associated with microtubules when they are present.

To assess the long-term effects of CKAP2 loss-of-function, cells were transfected with shRNA, and single cell clones were obtained. Depletion of CKAP2 protein was verified by Western blot analysis (Figure 1D). Subsequent experiments were performed with two single cell clones (CKAP2_8 and CKAP2_12). We have also shown that long-term reduction of CKAP2 expression has little to no effect on cell proliferation (Figure 1E and Figure S2). In addition, we observed that decreased expression of CKAP2 mediated by siRNA showed a very limited effect on cellular viability (Figure S3).

### Suppression of CKAP2 Increases Spindle Pole Defects

**Figure 2:**
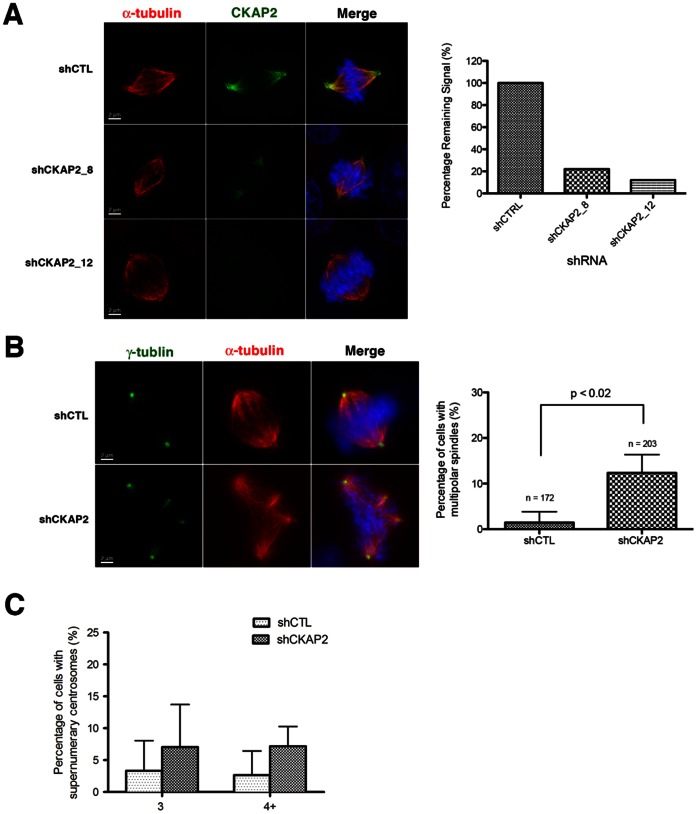
CKAP2 is required for bipolar spindle assembly. (A) Representative images of control (shCTL) and CKAP2-depleted (shCKAP2) cells co-immunstained with CKAP2 (green), α-tubulin (red), and merged with DAPI (blue) (Scale bar: 2 µm). The data is presented as the mean green intensity per experiment group. One hundred images were analyzed per experimental group. (B) Mitotic cells in asysnchronous populations of control (shCTL) and CKAP2-depleted (shCKAP2) cells were analyzed for mitotic defects by co-immunostaining with γ-tubulin (green) and α-tubulin (red). Representative images of multipolar spindles were observed CKAP2-depleted cells. Over 100 spindles per experimental group were analyzed in two independent experiments. The results are presented as mean ± SD (Scale bar: 2 µm). P-values were determined using the Student’s t-test. (C) Thirty nuclei were analyzed to assess the percentage of cells with supernumerary centrosomes in CKAP2-depleted cells compared to controls.

Data obtained from CKAP2 expression and localization analyses suggested that the effects of silencing this gene would be most obvious in mitotic cells, particularly affecting the mitotic spindle. Thus, we assessed the integrity of the mitotic spindle by immunofluorescence with α-tubulin as a marker for the mitotic spindle and γ-tubulin as a marker for centrosomes. Immunofluorescence assays confirmed the reduction in protein expression with a 78% and 88% decrease in signal intensity at the mitotic spindle in clones CKAP2_8 and CKAP2_12, respectively (Figure 2A). We found that reduction of CKAP2 expression resulted in a significant increase in cells with multipolar spindles (from 3% to 13% of cells, P<0.02) (Figure 2B). Consequently, we also observed an increase (5–10%) in supernumerary centrosomes in CKAP2-depleted cells, although the difference was not statistically significant (Figure 2C). An interesting observation was a reduced frequency of centrosome clustering, commonly found in cells with multiple centrosome, in CKAP2-depleted cells when compared to control cells (data not shown).

**Figure 3:**
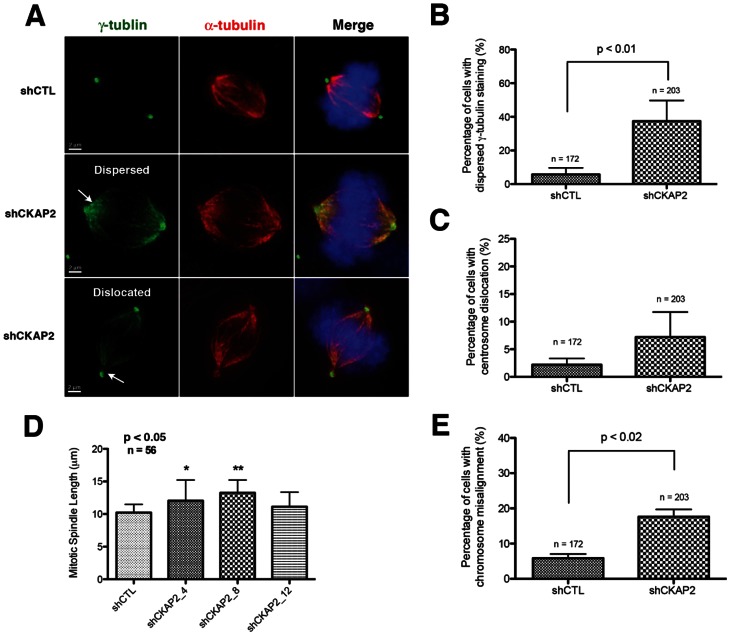
Spindle pole defects in CKAP2-depleted cells. (A) Mitosis in an asynchronous population of control (shCTL) and CKAP2-depleted (shCKAP2) cells were co-immunostained with γ-tubulin (green), α-tubulin (red), and merged with DAPI (blue). The dispersal of γ-tubulin away from the centrosome and dislocation of the centrosome from the spindle pole was analyzed in 200 cells per experimental group in two independent experiments. Representative images for each experimental group and the mitotic defect are shown (Scale bar: 2 µm). Representative images for each experimental group are shown. (B) Quantification of the cells with dispersed γ-tubulin was presented as mean ± SD. P-values were determined using the Student’s t-test. (C) Quantification of the cells with the centrosome dislocated from the spindle pole was presented as mean ± SD. P-values were determined using the Student’s t-test (D) Spindle length measured in 50 mitotic cells with bipolar spindles in both controls and CKAP2-depleted cells is shown. P-value was determined by Student’s t-test. (E) Analysis of the number of misaligned chromosomes in bipolar metaphases shows statistical significant difference between control and CKAP2-depleted cells. More than 200 cells were counted per condition. P-value was determined by Student’s t-test.

Using γ-tubulin as a marker for centrosomes, we then assessed the integrity of spindle poles in the absence of CKAP2. We observed a significant increase of cells where the γ-tubulin signal was dispersed along the mitotic spindle (from 5% to 40%, P<0.01) (Figure 3A and 3B). While not statistically significant, we also detected an increase in the percentage of cells where one centrosome was dislocated from the spindle pole (from 3% to 8%) (Figure 3A and 3C). In order to examine spindle pole function, we assessed spindle length by measuring the distance between the two spindle poles. Data showed that there was an increase up to 5 µm in the distance between spindle poles when compared shCKAP2_4 and shCKAP2_8 transfected cells to control cells (P<0.05) (Figure 3D). This increase in the spindle length may result from changes in the distribution of forces across the spindle, as this phenotype was accompanied by an increase in misalignment of metaphase chromosomes (from 6% to 18%, P<0.02) (Figure 3E). Overall, these data suggest that despite spindle abnormalities, cells remain able to form functional mitotic spindles.

### CKAP2 is Crucial for Maintaining the Integrity of the Microtubule Nucleation Sites in Early Mitosis

**Figure 4:**
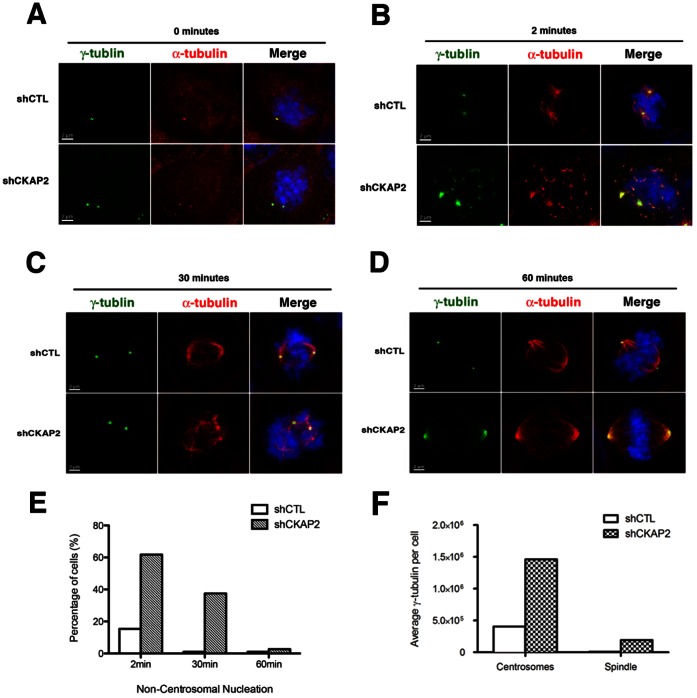
CKAP2 is required for anchoring of centrosome-nucleated microtubules to the spindle pole. (A) Nucleation was assessed after treating cells with 10 µg/ml nocodazole for 30 minutes and released into fresh media for 2, 30, and 60 minutes. shCTL and CKAP2-depleted cells were co-immunostained with γ-tubulin (green), Tyr-tubulin (red), and merged with DAPI (blue). (B) Two minutes post-nocodazole release, a cage-like structure was often observed in CKAP2-depleted cells. Representative images for each experimental group are shown. (C) Thirty minutes post-nocodazole release, microtubules are tethered at distinct poles, often with an increase cells with multipolar spindle poles in CKAP2-depleted cells. Representative images for each experimental group are shown. (D) Sixty minutes post nocodazole block, both the control and CKAP2-depleted cells have structured bipolar assembly. Representative images for each experimental group are shown. (E) Quantification of the shCTL cells with non-centrosomal α-tubulin staining at 2, 30, and 60 minutes is shown. Approximately 50 cells were counted per condition. (F) Measurements of the total γ-tubulin in both the centrosomes and the spindle pole area for CKAP2-depleted cells and controls. Y-axis indicates signal intensity units for γ-tubulin.

Because of the observed defects in γ-tubulin, in particular those resulting in signal dispersal along the mitotic spindle, we next examined to what extent CKAP2 was required for centrosome-nucleated microtubule formation and for microtubule stability. Asynchronous cells were treated with nocodazole for 30 minutes to depolymerize microtubules (Figure 4A). Cells were released from nocodazole for 2, 30, and 60 minutes and microtubule re-growth was analyzed in mitotic cells using an antibody (anti-α-tubulin YL1/2) specific to newly polymerized tubulin (so called Tyr-tubulin). Strikingly, we observed as early as two minutes after release an unusual phenotype consisting of dispersal of newly synthesized microtubule filaments that, in normal conditions, should be properly bound to the spindle pole. In CKAP2-depleted cells, around 60% of cells showed this cage-like pattern of dispersed nascent microtubules while we only identified this phenomenon in less than 20% of cells when transfected with an empty vector control (P<0.01) (Figure 4B and 4E). Nevertheless, 30 minutes post-nocodazole release, the percentage of cage-like spindles decreased to 40% and microtubule filaments began forming distinct poles (Figure 4C and 4E), and nearly 100% of cells displayed successful bipolar spindles at 60 minutes post-nocodazole release (Figure 4D and 4E). In addition, the total level of γ-tubulin was increased in CKAP2-depleted cells compared to control cells (Figure 4F). Further analysis showed that the centrosomal area (indicated by signal intensity) was also larger although it did not reach significance (Figure S4A). Moreover, we observed additional γ-tubulin, albeit a lesser amount, along the length of the spindle. This might indicate that more γ-tubulin is being recruited in CKAP2-depleted cells.

**Figure 5:**
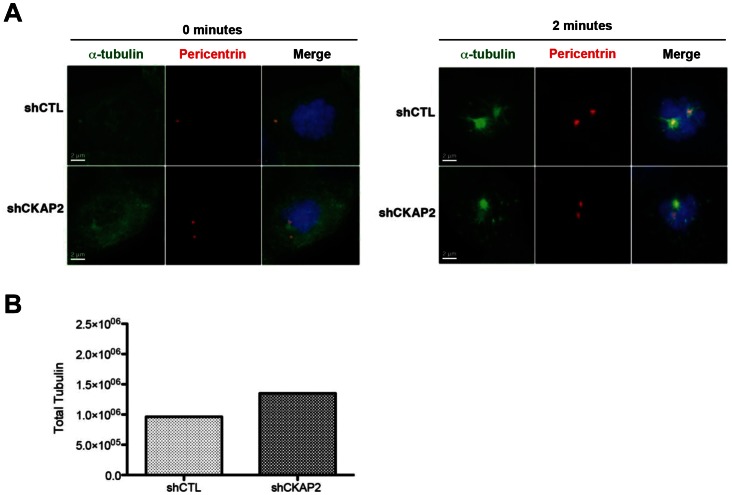
Centrosome nucleation capacity is unaffected in CKAP2-depleted cells. (A) Two minutes post-nocodazole release shCTL and CKAP2-depleted cells were co-immunostained with α-tubulin (DM1A) (green), pericentrin (red), and merged with DAPI (blue). One hundred cells with non-centrosomal tubulin staining were measured per experimental group. As already demonstrated, a cage-like structure was observed two minutes post-nocodazole release. Representative images for each experimental group are shown. (B) Nucleation capacity was determined by measuring the mean of α-tubulin fluorescence for both the control and CKAP2-depleted cells.

Immunostaining of pericentrin, an integral protein component of the centrosome, showed two prominent foci in the majority of CKAP2-depleted cells (Figure 5A), demonstrating that, despite the spindle pole dispersal, the centrosome remained intact. Furthermore, total microtubule measurements assessed by immunostaining against total tubulin showed that there was a slight increase in the amount of total polymerized α-tubulin (Figure 5B and Figure S4B and S4C), indicating that the total nucleation capacity of the centrosome is not reduced, but disorganized. The mechanism by which this increase in total tubulin polymer might occur has yet to be fully elucidated.

In order to investigate the possibility that CKAP2-depleted cells were utilizing a kinetochore- or chromatin-driven microtubule polymerization mechanism to overcome centrosomal nucleation difficulties, we assessed the localization of the dispersed microtubule filaments in relation to kinetochores. For this, we visualized Hec1, a kinetochore component, and α-tubulin using immunofluorescence. Results indicated that nascent microtubules were neither associated with nor nucleated from kinetochores, as there was no co-localization of α-tubulin signal to Hec1 (Figure S4D). Similarly, the presence of microtubule filaments that positioned outside chromatin boundaries disproved the hypothesis of a chromatin-directed microtubule polymerization. These data, together with the fact that CKAP2 is not normally found near the kinetochores or chromatin (Figure 1A and 1B), prompted us to conclude that CKAP2-depleted cells did not utilize the non-centrosomal nucleation mechanism to promote microtubule polymerization.

**Figure 6:**
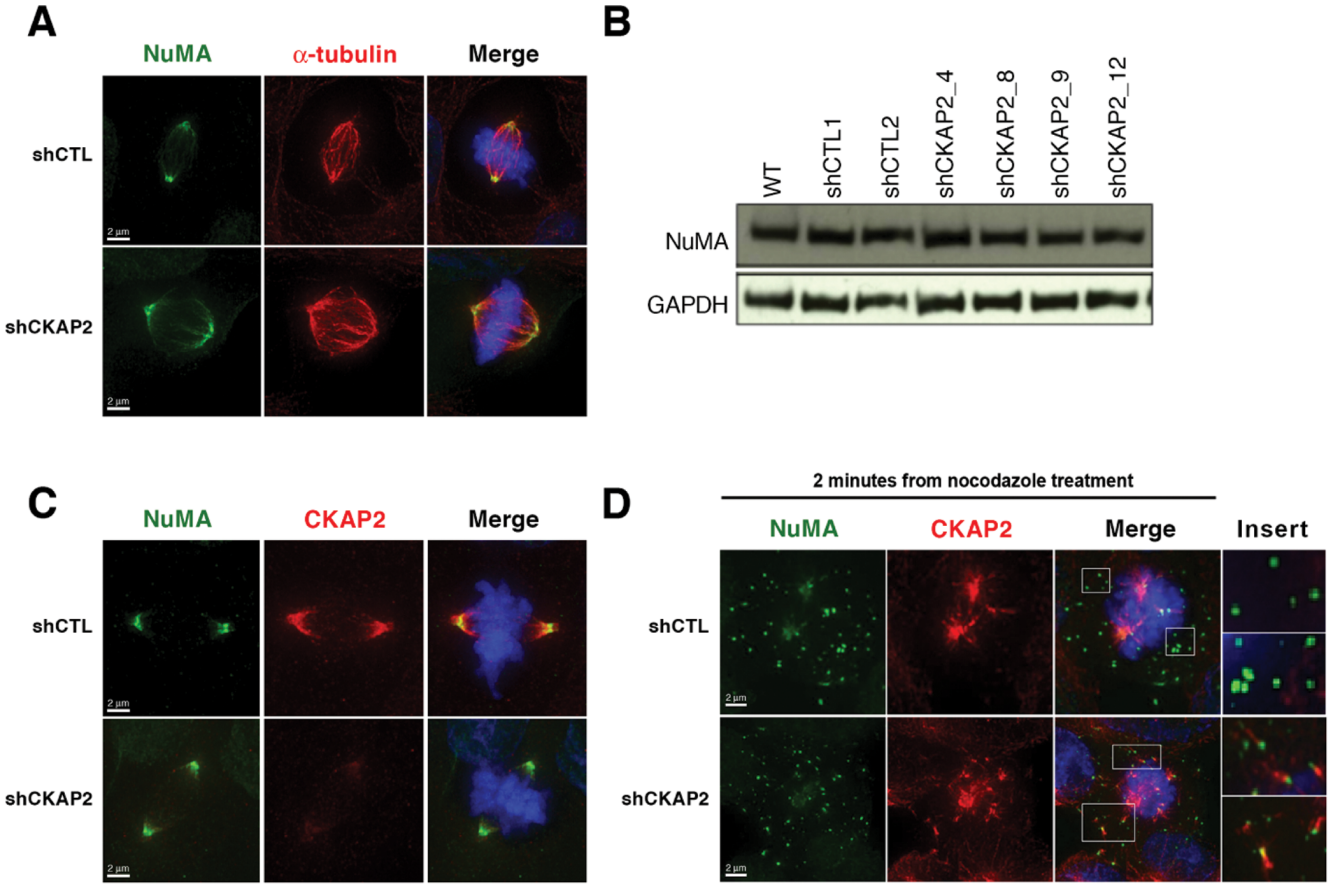
NuMA expression and localization is not affected by CKAP2-depletion. (A) shCTL and shCKAP2 transfected cells were co-immunostained with NuMA (green) and α-tubulin (red), and merged with DAPI (blue). The expression of NuMA was confined to the spindle pole. Representative metaphase images show that the localization of NuMA remains intact. (B) Immunoblot analysis with antibodies specific for NuMA and GAPDH showed that the amount of NuMA protein was maintained despite the silencing of CKAP2. (C) shCTL and CKAP2-depleted cells were co-immunostained with NuMA (green), CKAP2 (red), and merged with DAPI (blue) showing only partial overlay between the two protein although they both are located at the spindle pole. (D) shCTL and CKAP2-depleted cells were synchronized with nocodazole, and after two minutes post release cells were co-immunostained with NuMA (green) and α-tubulin (red). Co-localization of NuMA and α-tubulin is shown in the cage-like structures in CKAP2-depleted cells, but not in control cells.

One of the major proteins involved in cross-linking and positioning microtubule minus ends to the spindle poles is NuMA (Nuclear Mitotic Apparatus) [22]. To assess whether depletion of CKAP2 affected the positioning of NuMA, mitotic cells in asynchronous cell populations were analyzed for NuMA localization and expression. NuMA localized to the centrosomes and spindle poles in both control and CKAP2-depleted cells (Figure 6A), and Western blot analysis showed that the abundance of NuMA is equally maintained (Figure 6B). Although NuMA and CKAP2 both localize to the spindle pole, there was no complete overlay between the two proteins (Figure 6C). As expected, two minutes post-nocodazole treatment and release, both control and shRNA transfected cells showed a scattered distribution of NuMA, which may indicate that NuMA mobilizes to the spindle poles. However, in CKAP2-depleted cells where spindle pole dispersal was evident, we observed co-localization of nascent microtubules and NuMA two minutes post-nocodazole release (Figure 6D), confirming the finding that the nucleating centers are dispersed throughout the entire chromatin region. This might suggest a mechanism by which the dispersed microtubules are gathered to the spindle pole, and supports the interpretation that CKAP2 might be involved in the recruitment and maintenance of microtubule minus ends at the spindle poles.

### Silencing of CKAP2 Expression Results in a Higher Incidence of Merotelic Attachments, Anaphase Lagging Chromosomes, and Chromosomal Instability

**Figure 7:**
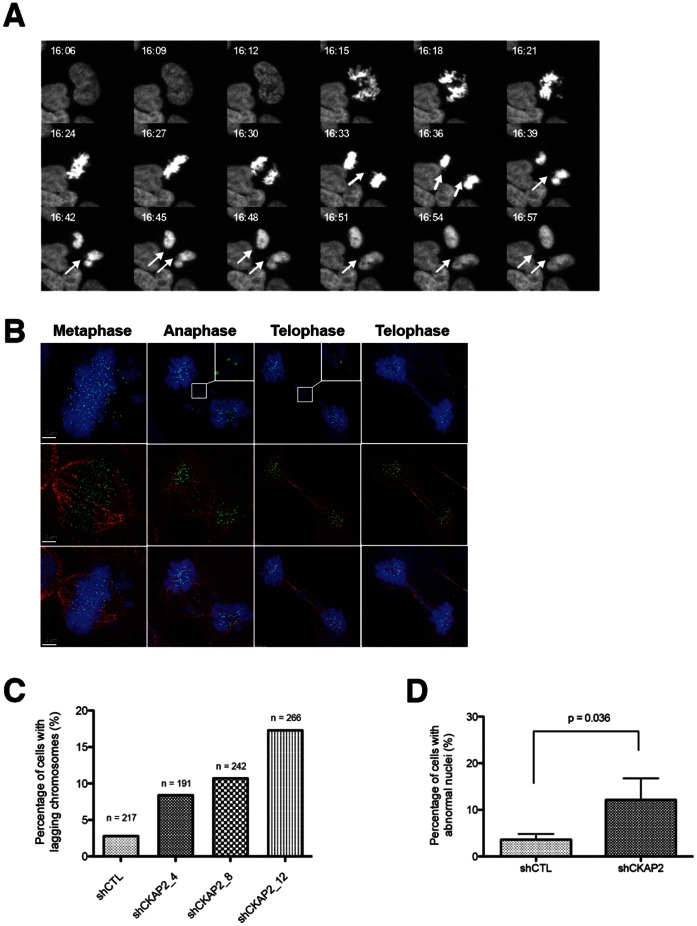
CKAP2-depleted cells show increased chromosome missegregation. (A) Control (shCTL) and CKAP2-depleted (shCKAP2) cells were transfected with histone H2B-Cherry constructs, selected with geneticin (G418), and analyzed with live-cell imaging. The movie shows CKAP2-depleted histone H2B-Cherry positive cells undergoing aberrant mitosis with chromosome missegregation resulting in two daughter nuclei with micronuclei. Arrows indicate lagging chromosomes and resultant micronuclei. (B) CKAP2-depleted cells were immunostained for Hec1 (green), α-tubulin (red), and merged with DAPI (blue). Cells with lagging chromosomes in anaphase and telophase were analyzed for merotelic attachments. Representative images of lagging chromosomes in anaphase and telophase are shown here. Magnified views emphasize merotelic attachments in lagging chromosomes. (C) The histogram represents the number of chromosome missegregation events for each histone H2B-Cherry positive experimental group. (D) Asynchronous shCTL and CKAP2-depleted cells were analyzed for evidence of chromosome missegregation, including micronuclei, nuclear blebs, and anaphase bridges. The results are plotted as the mean ± SD.

We next investigated how the observed spindle assembly defects affected chromosome segregation. Using an asynchronous population of cells, we monitored the incidence of anaphase lagging chromosomes, micronuclei, and nuclear blebs. By performing live-cell imaging using a histone H2B-Cherry construct, we observed a higher incidence of anaphase lagging chromosomes in CKAP2-depleted cells, which lead to the formation of micronuclei (Figure 7A). In order to further understand the cause of these segregation errors, we performed immunostaining for Hec1 and α-tubulin. First, we found that these anaphase lagging chromosomes displayed merotelic kinetochore-microtubule attachments (Figure 7B). Second, a higher incidence of anaphase lagging chromosomes in CKAP2-depleted cells was observed. In fact, in between 9% and 17% of cells from the clones with suppressed CKAP2 showed lagging chromosomes, which represented an increase of about 3-5-fold over the control cell population (Figure 7C). As a consequence of the elevated rate of missegregation, a significant increase of abnormal nuclear shapes, including micronuclei and nuclear blebs, was identified in a drug-free asynchronous cell population analysis when comparing CKAP2-depleted cells with control cells (P<0.05) (Figure 7D). Therefore, we concluded that depletion of CKAP2 promoted merotelic kinetochore-microtubule attachments that resulted in anaphase lagging chromosomes and increased chromosomal instability.

**Figure 8:**
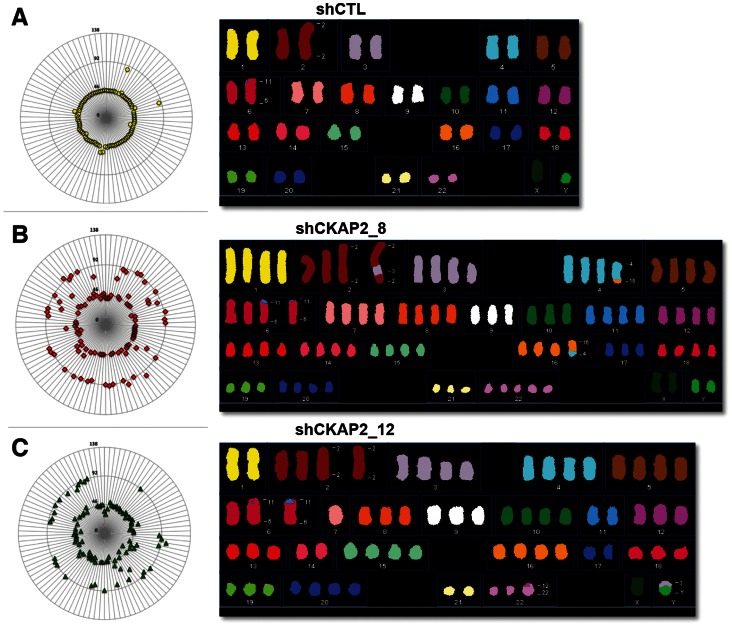
CKAP2 plays a role in maintaining chromosome stability. (A–C) Mitotic cells were treated with colcemid in order to obtain metaphase spreads. Chromosome content was determined by counting the individual chromosomes in at least 100 metaphases. The results are presented as radial plots, where the concentric circle represents the relative ploidy and each symbol represents an individual cell. In parallel, here indicated are karyotypes analyzed by SKY showing the increased level of aneuploidy and chromosome instability in CKAP2-depleted cells.

Analysis of the karyotype of CKAP2-depleted cells revealed a higher incidence of chromosomal heterogeneity in the clones compared to control cells. As illustrated by the radial plots in Figure 8, the karyotype of control cells showed a perfect distribution of the expected modal number (2N = 46) for DLD-1 cells. However, suppression of CKAP2 in the clones resulted in an extraordinary increase of aneuploidy (22.6% polyploid cells for clone CKAP2_8 and 36.2% for clone CKAP2_12 compared to 2.2% for control cells). In addition, spectral karyotype (SKY) analysis for a representative subset of cells in each clone confirmed ongoing patterns of aneuploidy and showed de novo clonal structural chromosome aberrations, corroborating the elevated incidence of chromosomal instability in these cells.

## Discussion

In the present study, we have characterized the cellular function of the cytoskeleton-associated protein 2 (CKAP2), also known as tumor-associated microtubule-associated protein 2 (TMAP), as a component of the spindle pole apparatus involved in maintaining proper microtubule nucleation sites. Our data confirm that CKAP2 co-localizes with microtubules in the mitotic spindle [23], [24], particularly at the spindle pole [21], where it appears to play its major role in mitotic spindle assembly.

A major function of the spindle pole is to bundle new centrosome-nucleated mitotic microtubules and anchor microtubule minus ends after the nuclear envelope breakdown at the onset of mitosis occurs.

Here, we demonstrate that CKAP2-depleted cells showed a decrease in spindle pole integrity and concomitant increase of organizational spindle defects, most notably including multipolar spindles and dispersion of γ-tubulin from the centrosome. In addition, we identified an increase of γ- and α-tubulin when CKAP2 is absent. Despite this phenotype, the distribution and function of centrosomes remained intact. This observation prompted us to determine whether CKAP2 is required for microtubule nucleation. Analysis of the nucleation capacity of the centrosomes showed that nascent microtubules were dispersed across the chromatin region at early time points after the mitotic release. These dispersed microtubules gathered into specific poles and became bipolar at later time points (30 and 60 minutes), albeit with a temporary increase in the number of spindle poles. Further analysis indicated that depletion of CKAP2 increases both the microtubule-bound and centrosomal pool. However, technically it is difficult to clearly identify the centrosome in CKAP2-depleted cells, and thus we can only speculate that it is the centrosome area. Of course, it is still possible that there is an increase of γ-tubulin more at the microtubule surface in the spindle pole area. Non-centrosomal sites of γ-tubulin can represent novel sites of nucleation or capped microtubule minus ends that were released from the centrosome [25]. However, we failed to observe microtubule nucleation at or near the kinetochores and chromatin, therefore we conclude that chromosome-mediated nucleation is not responsible for the observed ectopic nascent microtubules. The increase in spindle intensity may indicate a slight increment in microtubule stabilization or enhanced microtubule nucleation, potentially via other proteins such as TACC or TPX2, both known to be potent microtubule nucleators besides γ-tubulin.

Microtubules forming from the centrosome do not remain tightly bound in early mitosis, but, nonetheless, they are anchored in the vicinity of the pericentriolar material (PCM) by a group of proteins that form the spindle pole matrix. Whether these microtubules come from newly polymerized microtubules detaching from the pole or whether they are fragmented microtubule minus ends is still undetermined [26]. In CKAP2-depleted cells, microtubules nucleated in early mitosis are not held in the vicinity of the PCM and thus dispersed across the nucleus. The presence of this phenotype strongly supports its subcellular localization within the spindle pole matrix.

One of the principal molecular components in tethering and focusing spindle microtubules at the poles is NuMA [27]. Depletion of NuMA results in aberrant spindles, pole fragmentation, and dissociation of the centrosome from already assembled spindles, resulting in splayed microtubule ends [22], [28], [29]. CKAP2 showed functional similarities with NuMA and, like NuMA, CKAP2 mitotic activity is also regulated by phosphorylation and dephosphorylation of residues near the C-terminal end [15], [30]. However, we have shown here that depletion of CKAP2 does not compromise the localization and expression of NuMA. In fact, the distribution of NuMA after microtubule depolymerization and re-growth was unaltered in CKAP2-depleted cells. Moreover, most CKAP2-depleted cells are still capable of forming bipolar spindles after nocodozale washout, which suggests that the absence of CKAP2 does not impede NuMA to cross-link microtubules and form the normal bipolar spindle. Perturbation of other well-known spindle proteins, such as Aurora A, TPX2, or ch-TOG, also compromises the integrity of the spindle pole, ultimately resulting in microtubule disorganization, multipolar spindles, and increased aberrant microtubule-kinetochore attachments [31]–[34]. Similar to NuMA, the localization and expression of TPX2 was not altered upon depletion of CKAP2 (Figure S5). These observations prompted us to assume that CKAP2 does not affect dynein-dependent transport of spindle pole organizing proteins. Nevertheless, CKAP2 depletion does increase multipolar spindles, largely due to multiple γ-tubulin foci. Hence, we propose that the dispersion of γ-tubulin could lead to the formation of additional spindle poles, as well as spindle pole defects.

Defects in kinetochore-microtubule and spindle-microtubule forces result in an increase in merotelic attachments [35], [36]. Merotelic attachments occur when a single kinetochore binds microtubules from two spindle poles instead of just one. Because merotelic kinetochore attachments are not detected by the mitotic checkpoint, cells with merotelic kinetochores can progress through mitosis [3]. Although most merotelically-oriented chromosomes segregate properly, a fraction of them remain at the metaphase plate while the other chromosomes move to the poles [37]. Recently, it has been suggested that segregation errors occur as a consequence of cells passing through a transient multipolar spindle intermediate that triggers the formation of merotelic attachments [38]. The spindle defects observed in CKAP2-depleted cells suggest that these cells undergo a transient multipolar spindle state, which would explain the increase in merotelic kinetochore attachments, forming anaphase lagging chromosomes (Figure S6).

Previous studies in mouse and human fibroblasts have shown that centrosome separation and establishment of the spindle apparatus were not noticeably affected in CKAP2-depleted cells [14]. The same authors also concluded that accumulation of chromosomes at the metaphase plate appeared largely unaffected and suggested that functional aspects of the microtubule spindle apparatus remained mostly unaltered. Our data provide further evidence that the absence of CKAP2 does not permanently affect the spindle structure and may not be essential to maintain cellular viability. We showed that although cells display a large number of extra microtubule organizing centers at early time points after nocodazole washout, CKAP2 appears largely dispensable for the establishment of the bipolar spindle poles at later time-points. One possible explanation is that spindle pole/PCM proteins exhibit functional redundancy allowing proper bipolar spindle assembly even in the absence of certain components (e.g., CKAP2). However, despite the fact that the mitotic spindle remains functional, our data suggest that CKAP2-depleted cells exhibit a delay in organizing the microtubule nucleation sites in early mitosis and this delay may result in transient geometric defects of the mitotic spindle. While other mechanisms might be considered, we find this explanation appropriate as it combines the defect/delay observed in microtubule nucleation sites following nocodazole washout in CKAP2-depleted cells with the increased rate of chromosome missegregation and nuclear malformations (Figure 6C and 6D). Surprisingly, we detected a substantial subpopulation of cells with a near-tetraploid karyotype. As we did not observe systematic failure of cytokinesis and most of the cells only have two centrosomes, we speculate that an early mitotic slippage event is the mechanism by which these cells become polyploid. It has been argued that tetraploidy might represent an intermediate on the route to aneuploidy [39], [40]. In addition to alterations in the ploidy, we have also identified the formation of clonal structural chromosome rearrangements, indicative of high levels of chromosomal instability [41].

In conclusion, we provide clues to elucidate the cellular mechanism by which CKAP2 regulates proper chromosome segregation. CKAP2 is involved maintaining the integrity of microtubule nucleation sites in early mitosis to accurately form the spindle poles. Although there is apparently enough redundancy to ensure spindle formation and chromosome segregation, we propose that CKAP2 depletion increases the formation of transient multipolar spindles, likely due to a reduction in the ability to cluster centrosomes and additional spindle poles. This geometric defect allows for greater accessibility to kinetochores, resulting in an increase of aberrant microtubule-kinetochore attachments, a higher frequency of chromosome missegregation, and ultimately, chromosomal instability.

## Supporting Information

Figure S1 **CKAP2 expression is restricted to mitosis.** (A) Wild-type DLD1 cells were synchronized in mitosis with 100 ng/mL nocodazole for 16 hours and released for the indicated time points (1, 3, and 5 hours). The cells were harvested and analyzed by immunoblot with antibodies specific for CKAP2, cyclin B1, phospho-Histone H3, and GAPDH. (B) Progression from mitotic release through the cell cycle was verified by synchronizing wild-type cells with nocodazole as previously noted and released for the indicated time points. Cells were harvested, stained with propidium iodide and analyzed by FACS.
(TIF)

Figure S2 **CKAP2-depletion does not influence cell viability or cause an accumulation of cells in mitosis.** (A) Cell viability in shRNA transfected cells was analyzed by measuring the metabolic activity of shCTL and shCKAP2 cells 96 hours after plating. This histogram represents the remaining viable cells for each experimental group for six technical replicates. (B) Asynchronous shCTL and shCKAP2 cells were stained with propidium iodide and the DNA content was analyzed by FACS. The phases of cell cycle, G1, S, and G2/M, were determined based on 2N and 4N DNA content.
(TIF)

Figure S3 **Depletion of CKAP2 does affect cell viability in human colorectal cancer cell line DLD-1.** (A) DLD1 cells were transfected with control (siCTL) or CKAP2 (siCKAP2). Seventy-two hours later, RNA was extracted for qRT-PCR analysis. (B) Ninety-six hours post siRNA transfection, cells were harvested for immunoblot analysis with antibodies specific to CKAP2 and GAPDH. (C) Cell viability was analyzed by measuring the metabolic activity of siCTL and siCKAP2 cells 96 hours post siRNA transfection. The histogram represents the percentage of remaining viable cells relative to shCTL for each experimental group for six biological replicates. (D) Apoptosis was measured by costaining siCTL and siCKAP2 cells 72 hours post siRNA transfection with Annexin-V (x-axis) and 7-AAD (y-axis) and analyzed by FACS [negative control (untreated; top left), positive control (All Star Death; top right), siCKAP2 (bottom left and right).
(TIF)

Figure S4 **Centrosome nucleation capacity is unaffected in CKAP2-depleted cells.** (A) Plot showing intensity signal for total centrosome area stained with γ-tubulin (B) Total tubulin was analyzed for 100 cells thirty minutes post-nocodazole release by measuring the mean fluorescence intensity for α-tubulin DM1A staining. (C) Total tubulin was analyzed for 100 cells sixty minutes post-nocodazole release by measuring the mean fluorescence intensity for α-tubulin DM1A staining. (D) Two minutes post-nocodazole release, cells were co-immunostained with the kinetochore protein Hec1 (green), α-tubulin (red), and merged with DAPI (blue) to determine the presence of chromosome-directed nucleation. Co-localization of Hec1 and α-tubulin signals was analyzed in control and CKAP2-depleted cells. Representative images for each experimental group are shown.
(TIF)

Figure S5 **CKAP2 depletion does not affect the expression and localization of microtubule associated protein, TPX2.** (A) Control (shCTL) and CKAP2-depleted (shCKAP2) cells were immunostained with TPX2 (green), α-tubulin (red) and merge with DAPI (blue). Representative images for each experimental group are presented. (B) Mitotic cells in shCTL and shCKAP2 populations were enriched by nocodazole treatment for 16 hours and harvested for immunoblot analysis with antibodies specific for TPX2 and GAPDH.
(TIF)

Figure S6 **Cellular mechanism of action of CKAP2.** Absence of CKAP2 results in transient multipolar spindles, which in turn resulted in merotelic attachments, segregation errors, and chromosome instability.
(TIF)
