# Supplementary figures and images for: CKAP2 Ensures Chromosomal Stability by Maintaining the Integrity of Microtubule Nucleation Sites
Source: PLoS One. 2013 May 30;8(5):e64575. doi: 10.1371/journal.pone.0064575 (PMC3667829; doi:10.1371/journal.pone.0064575)

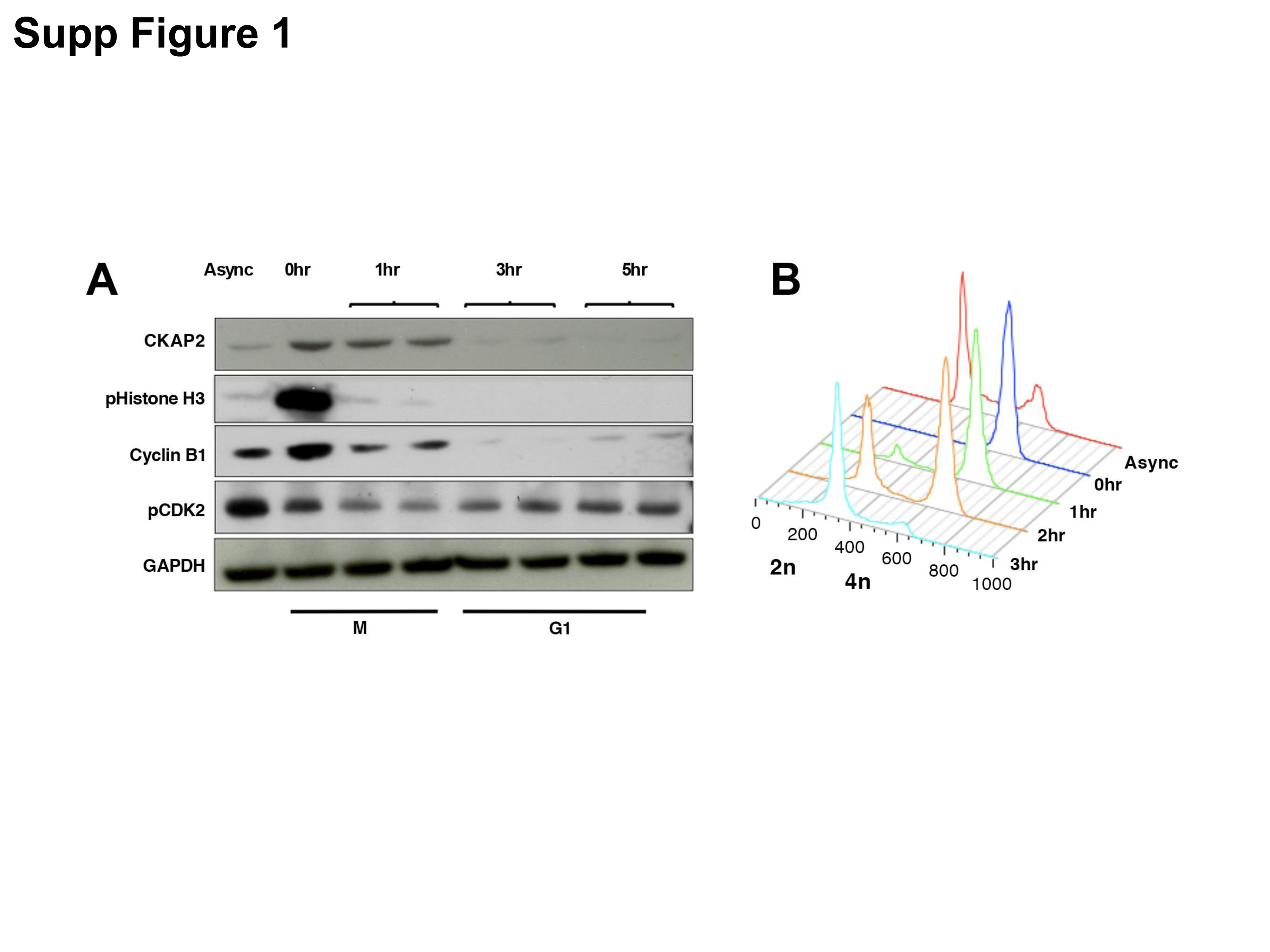

Supplement: Figure S1 — CKAP2 expression is restricted to mitosis. (A) Wild-type DLD1 cells were synchronized in mitosis with 100 ng/mL nocodazole for 16 hours and released for the indicated time points (1, 3, and 5 hours). The cells were harvested and analyzed by immunoblot with antibodies specific for CKAP2, cyclin B1, phospho-Histone H3, and GAPDH. (B) Progression from mitotic release through the cell cycle was verified by synchronizing wild-type cells with nocodazole as previously noted and released for the indicated time points. Cells were harvested, stained with propidium iodide and analyzed by FACS. (TIF) [file pone.0064575.s001.tif]

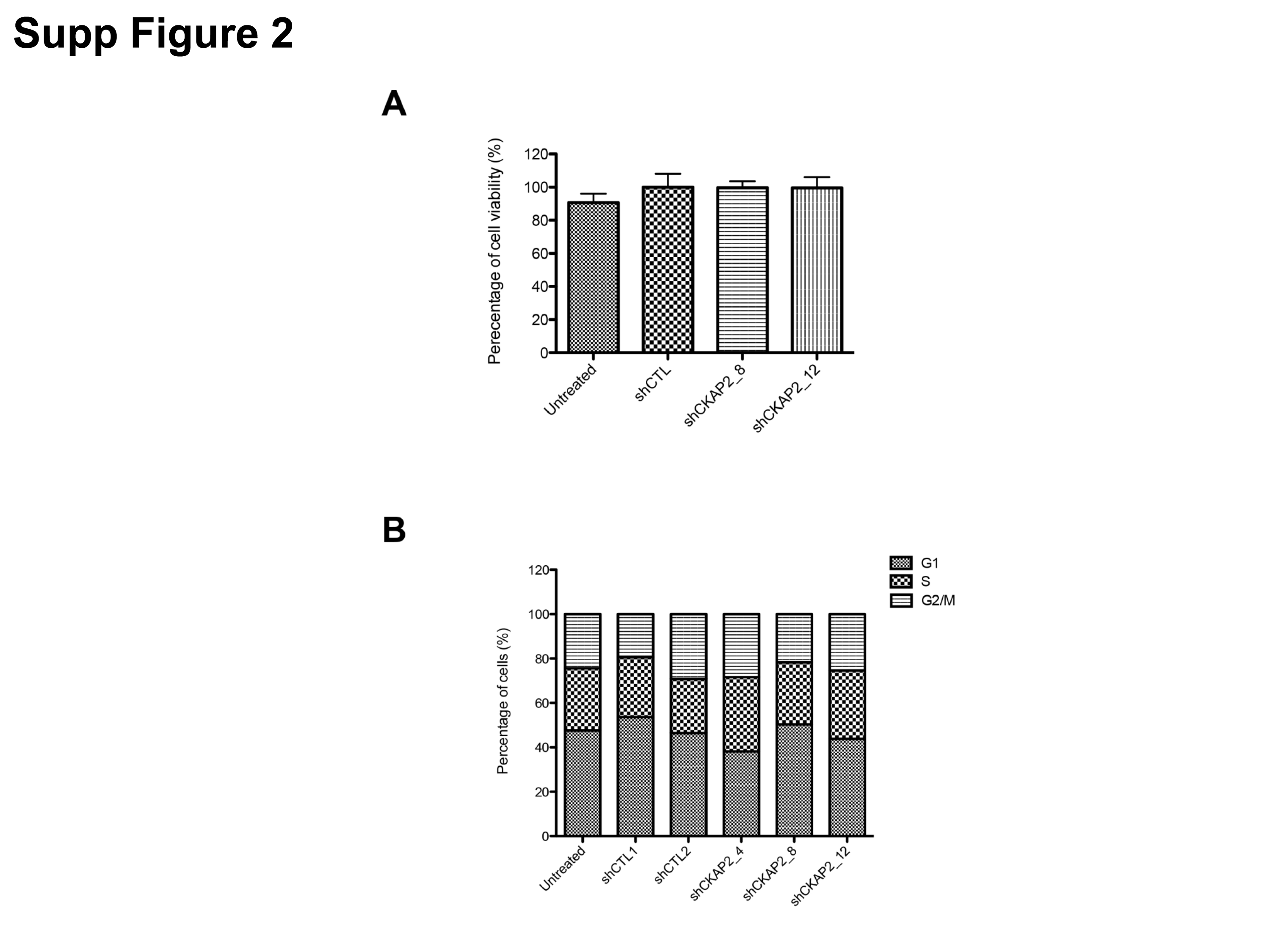

Supplement: Figure S2 — CKAP2-depletion does not influence cell viability or cause an accumulation of cells in mitosis. (A) Cell viability in shRNA transfected cells was analyzed by measuring the metabolic activity of shCTL and shCKAP2 cells 96 hours after plating. This histogram represents the remaining viable cells for each experimental group for six technical replicates. (B) Asynchronous shCTL and shCKAP2 cells were stained with propidium iodide and the DNA content was analyzed by FACS. The phases of cell cycle, G1, S, and G2/M, were determined based on 2N and 4N DNA content. (TIF) [file pone.0064575.s002.tif]

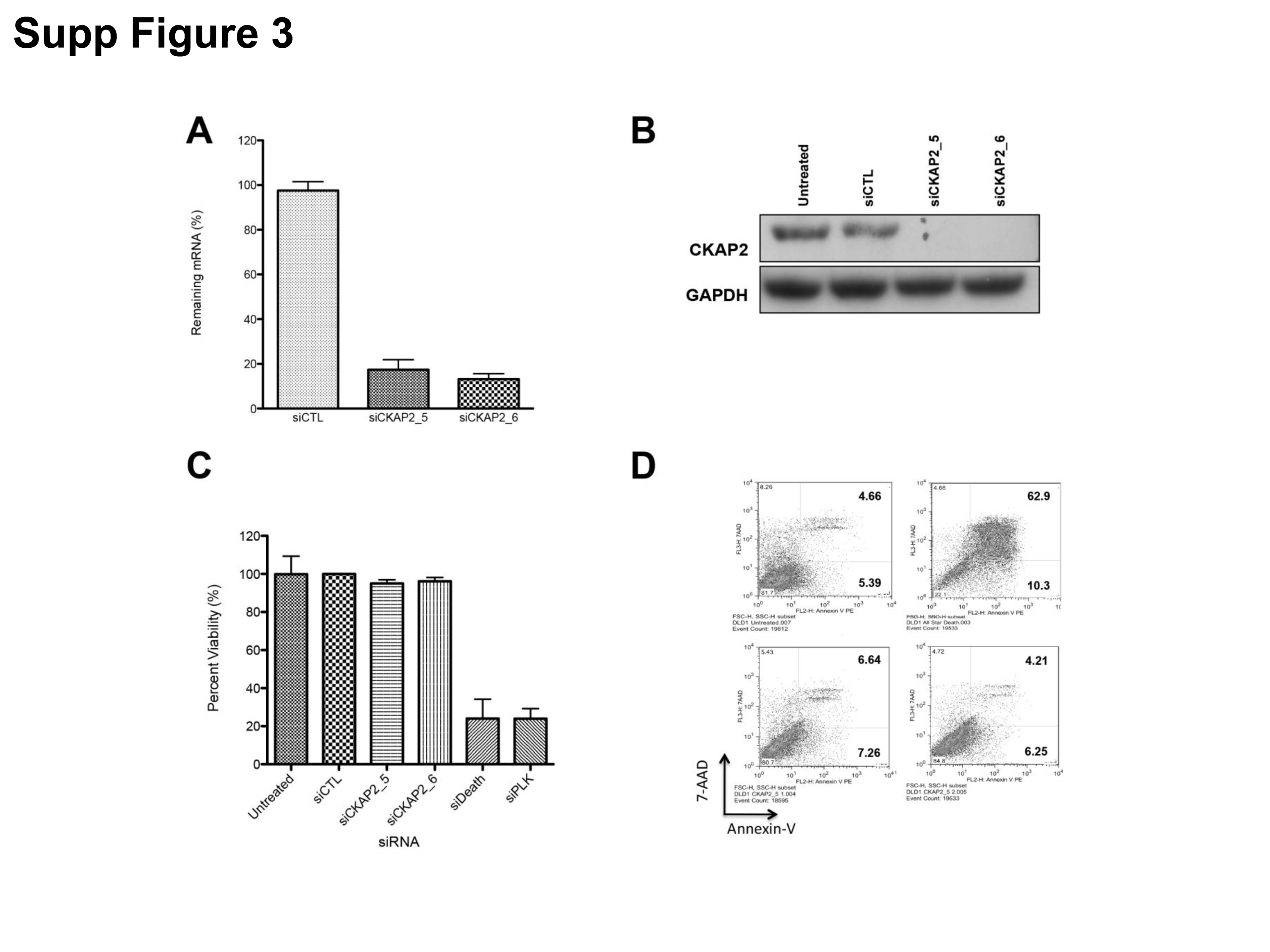

Supplement: Figure S3 — Depletion of CKAP2 does affect cell viability in human colorectal cancer cell line DLD-1. (A) DLD1 cells were transfected with control (siCTL) or CKAP2 (siCKAP2). Seventy-two hours later, RNA was extracted for qRT-PCR analysis. (B) Ninety-six hours post siRNA transfection, cells were harvested for immunoblot analysis with antibodies specific to CKAP2 and GAPDH. (C) Cell viability was analyzed by measuring the metabolic activity of siCTL and siCKAP2 cells 96 hours post siRNA transfection. The histogram represents the percentage of remaining viable cells relative to shCTL for each experimental group for six biological replicates. (D) Apoptosis was measured by costaining siCTL and siCKAP2 cells 72 hours post siRNA transfection with Annexin-V (x-axis) and 7-AAD (y-axis) and analyzed by FACS [negative control (untreated; top left), positive control (All Star Death; top right), siCKAP2 (bottom left and right). (TIF) [file pone.0064575.s003.tif]

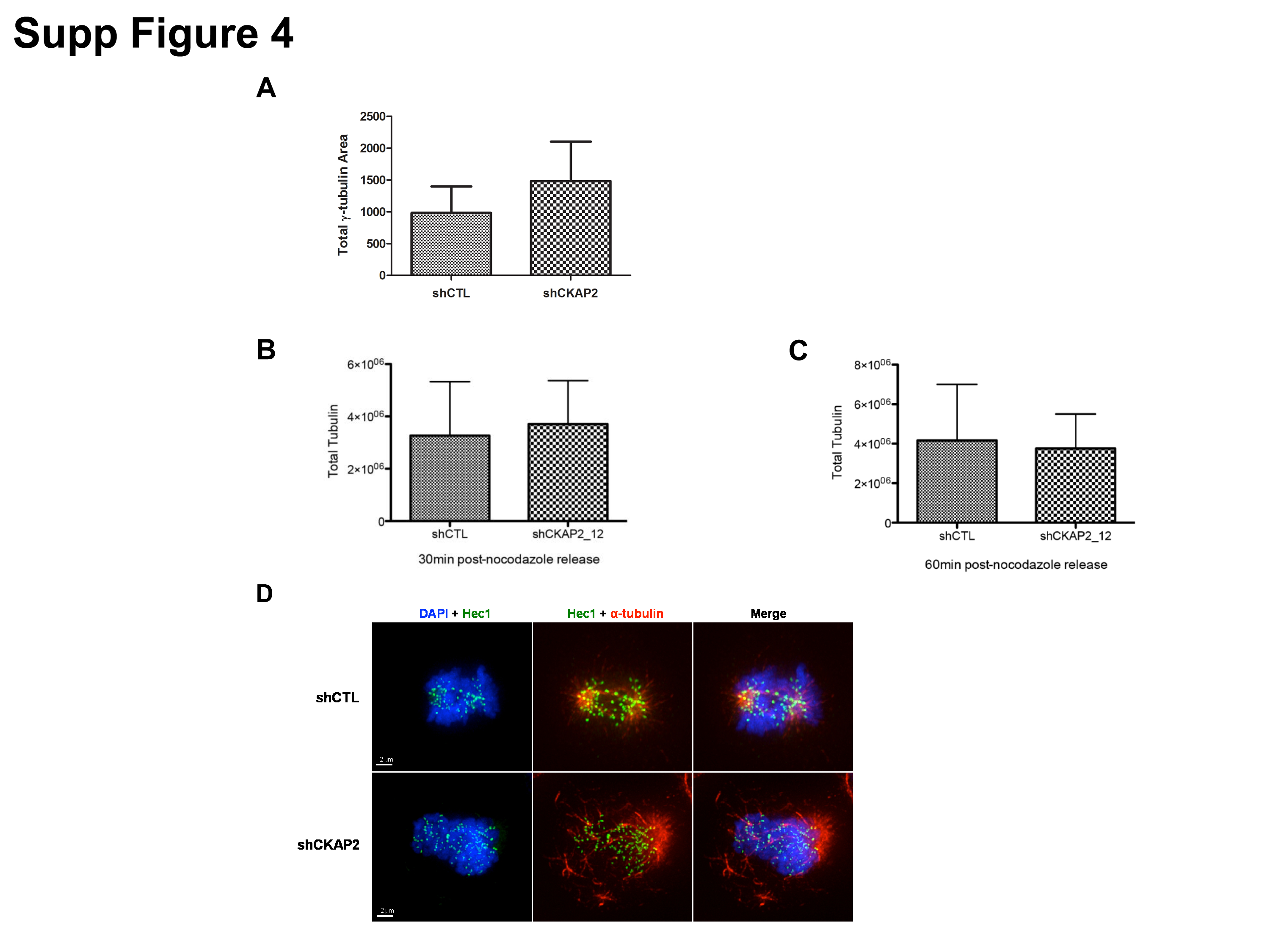

Supplement: Figure S4 — Centrosome nucleation capacity is unaffected in CKAP2-depleted cells. (A) Plot showing intensity signal for total centrosome area stained with γ-tubulin (B) Total tubulin was analyzed for 100 cells thirty minutes post-nocodazole release by measuring the mean fluorescence intensity for α-tubulin DM1A staining. (C) Total tubulin was analyzed for 100 cells sixty minutes post-nocodazole release by measuring the mean fluorescence intensity for α-tubulin DM1A staining. (D) Two minutes post-nocodazole release, cells were co-immunostained with the kinetochore protein Hec1 (green), α-tubulin (red), and merged with DAPI (blue) to determine the presence of chromosome-directed nucleation. Co-localization of Hec1 and α-tubulin signals was analyzed in control and CKAP2-depleted cells. Representative images for each experimental group are shown. (TIF) [file pone.0064575.s004.tif]

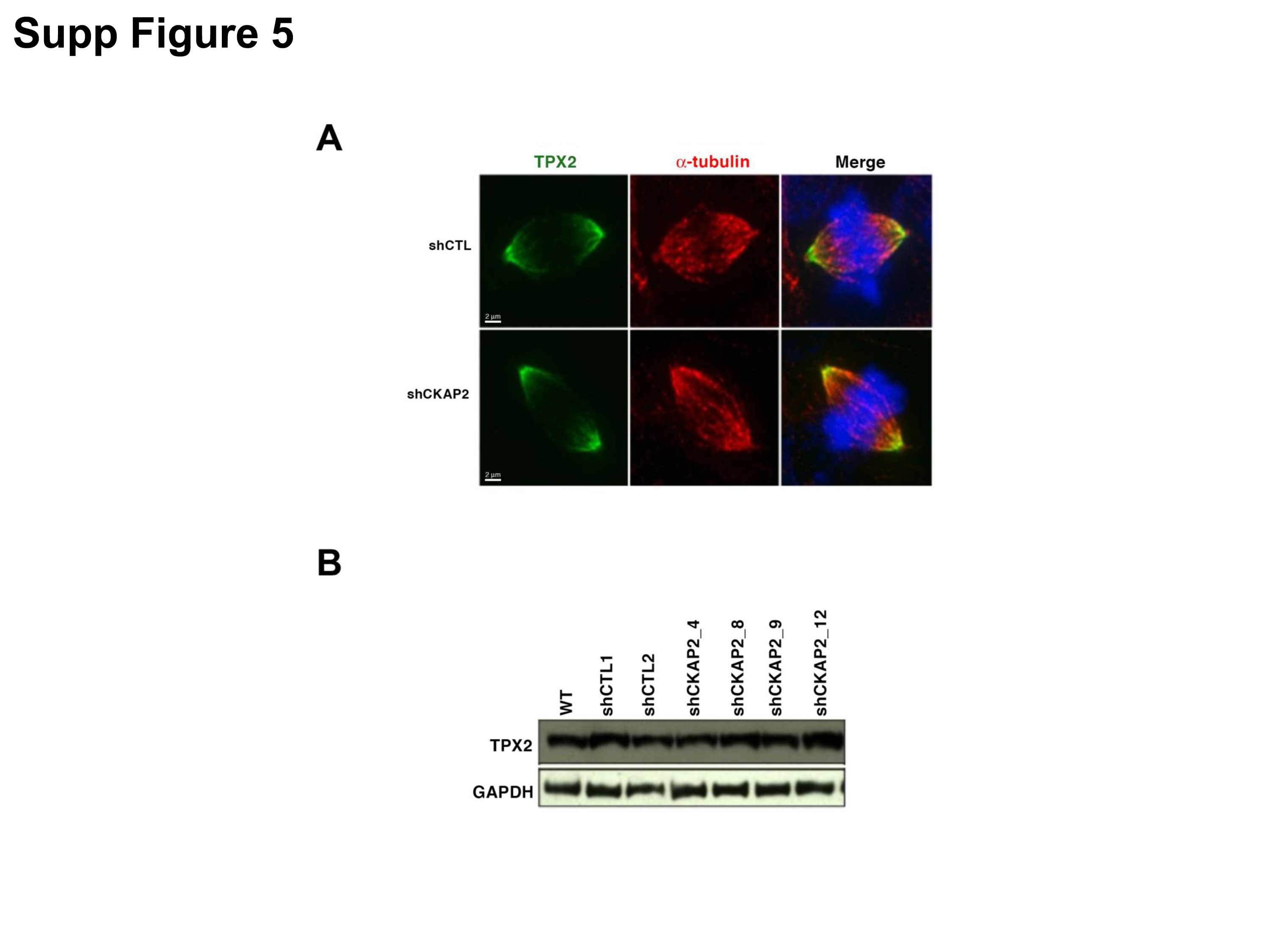

Supplement: Figure S5 — CKAP2 depletion does not affect the expression and localization of microtubule associated protein, TPX2. (A) Control (shCTL) and CKAP2-depleted (shCKAP2) cells were immunostained with TPX2 (green), α-tubulin (red) and merge with DAPI (blue). Representative images for each experimental group are presented. (B) Mitotic cells in shCTL and shCKAP2 populations were enriched by nocodazole treatment for 16 hours and harvested for immunoblot analysis with antibodies specific for TPX2 and GAPDH. (TIF) [file pone.0064575.s005.tif]

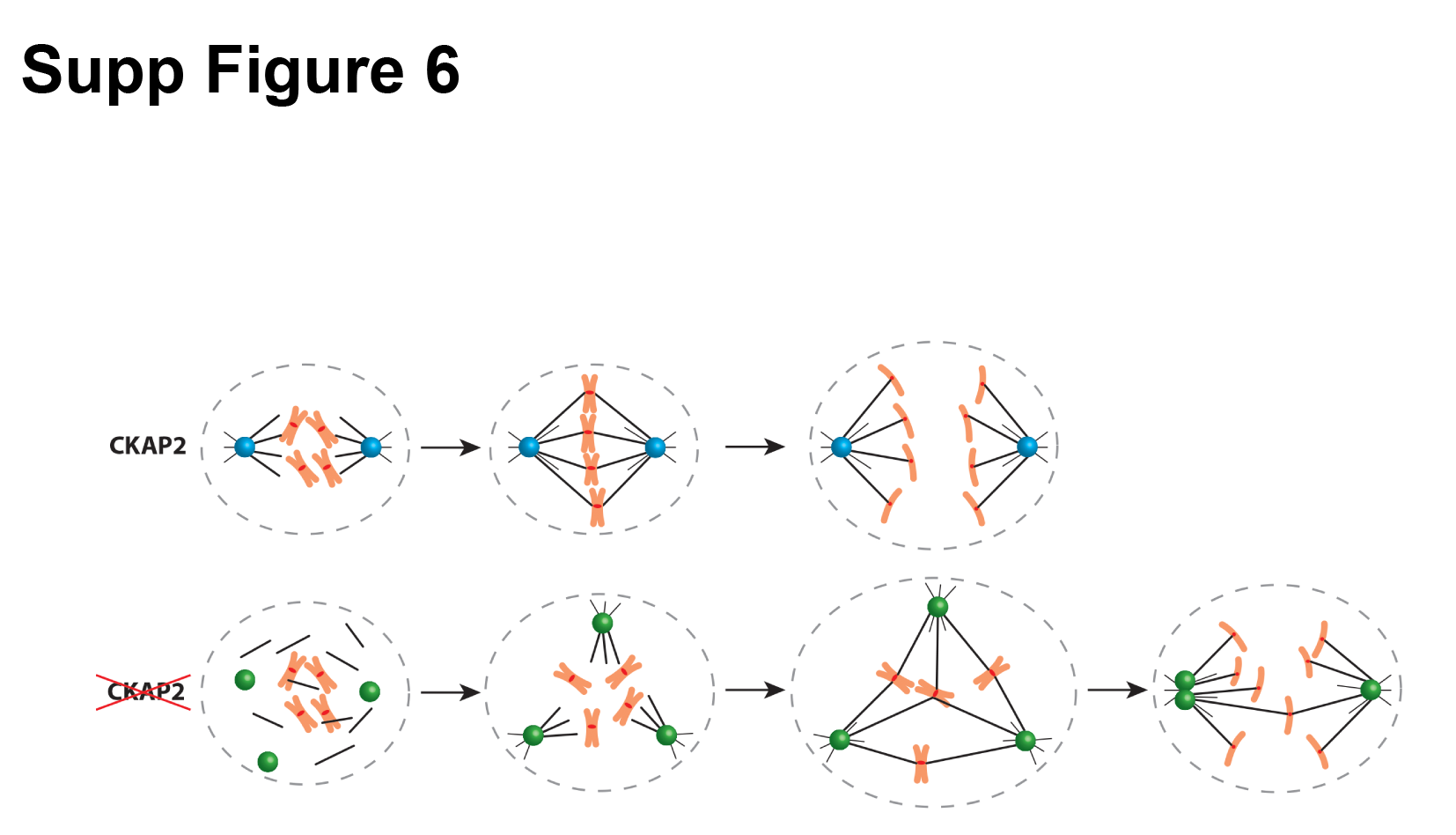

Supplement: Figure S6 — Cellular mechanism of action of CKAP2. Absence of CKAP2 results in transient multipolar spindles, which in turn resulted in merotelic attachments, segregation errors, and chromosome instability. (TIF) [file pone.0064575.s006.tif]
